# Indigenous mental healthcare and human rights abuses in Nigeria: The role of cultural syntonicity and stigmatization

**DOI:** 10.3389/fpubh.2023.1122396

**Published:** 2023-06-23

**Authors:** Adegboyega Ogunwale, Babatunde Fadipe, Oladayo Bifarin

**Affiliations:** ^1^Neuropsychiatric Hospital, Aro, Abeokuta, Abeokuta, Nigeria; ^2^Institute of Psychiatry, Psychology & Neuroscience, King's College London, London, United Kingdom; ^3^Lagos University Teaching Hospital, Lagos, Nigeria; ^4^School of Nursing and Advanced Practice, Faculty of Health, Liverpool John Moores University, Liverpool, North West England, United Kingdom; ^5^Mersey Care NHS Foundation Trust, Liverpool, United Kingdom

**Keywords:** indigenous, mental, healthcare, Nigeria, human rights

## Abstract

**Background:**

Indigenous mental healthcare using traditional non-western methods termed “unorthodox approaches” has been observed in Nigeria historically. This has been largely due to a cultural preference for spiritual or mystical rather than biomedical formulations of mental disorder. Yet, there have been recent concerns about human rights abuses within such treatment settings as well as their tendency to perpetuate stigmatization.

**Aim:**

The aim of this review was to examine the cultural framework for indigenous mental healthcare in Nigeria, the role of stigmatization in its utilization and interrogate the issues of human rights abuses within a public mental health context.

**Methods:**

This is a non-systematic narrative review of published literature on mental disorders, mental health service utilization, cultural issues, stigma, and indigenous mental healthcare. Media and advocacy reports related to human rights abuses in indigenous mental health treatment settings were also examined. International conventions on human rights and torture, national criminal legislation, constitutional provisions on fundamental rights and medical ethics guidelines relevant to patient care within the country were examined in order to highlight provisions regarding human rights abuses within the context of care.

**Results:**

Indigenous mental healthcare in Nigeria is culturally syntonic, has a complex interaction with stigmatization and is associated with incidents of human rights abuses especially torture of different variants. Three systemic responses to indigenous mental healthcare in Nigeria include: orthodox dichotomization, interactive dimensionalization, and collaborative shared care. Conclusions: Indigenous mental healthcare is endemic in Nigeria. Orthodox dichotomization is unlikely to produce a meaningful care response. Interactive dimensionalization provides a realistic psychosocial explanation for the utilization of indigenous mental healthcare. Collaborative shared care involving measured collaboration between orthodox mental health practitioners and indigenous mental health systems offers an effective as well as cost-effective intervention strategy. It reduces harmful effects of indigenous mental healthcare including human rights abuses and offers patients a culturally appropriate response to their problems

## 1. Introduction

Mental disorders appear to be on the rise all over the world and will cost the global economy up to $16 trillion in 20 years (2010–2030) if urgent steps are not taken (1). Psychosocial problems of COVID-19 (present and future) have complicated the mix. About $147 billion in investment is required to scale up treatments such as psychosocial counseling and antidepressant medication (2). In addressing the critical concern presented by these disorders, a multi-faceted approach to healthcare must be adopted.

Mental healthcare using traditional non-western methods termed “unorthodox approaches” has been observed in Nigeria and elsewhere for decades (3–7). This has been largely due to a cultural preference for spiritual rather than biomedical formulations of mental disorder (7–10). Other factors driving this tendency have included lack of access to care, out-of-pocket healthcare payments, poverty, poorly planned services, shortage of healthcare personnel and other resources, nearness of these facilities to the community and their shared belief with patients regarding the cause and treatment of mental disorder (4, 11–13) as well as stigma associated with mental illness. The existence and impact of stigma on the mentally ill in particular has been well studied (14, 15).

However, recent developments in Nigeria have signposted significant human rights abuses in non-orthodox mental healthcare settings in Nigeria (16). These facilities have been termed, variously, as “torture homes,” owing to victims’ accounts of physical, sexual and psychological abuse (17). Many of such facilities are run by faith and traditional healers with some adopting eclectic or syncretic approaches. Syncretic approaches refer to those who combine cultural methods with faith-based treatments.

Yet, research done in Nigeria and elsewhere indicates that these facilities are not just endemic but culturally syntonic. A recent study surveying complementary and alternative mental health treatment providers indicated their ubiquity in African settings with their capacity to provide admission services sometimes above the capacity of conventional hospitals (18). As well, the harmony of complementary and traditional medicine with cultural practices has long been recognized globally (19).

Given this background, this paper highlights three observable responses to this multi-faceted phenomenon. An initial response to the problem is that of outright prohibition of non-orthodox practice or a dichotomization between the orthodox and the non-orthodox. A second response is that of interactive dimensionalization where both forms of care occur in a cultural continuum. A third dimension is collaboration which has now been termed “collaborative shared care” (4).

This paper examines these constructs of public mental healthcare from a human rights and medico-legal perspective. This inquiry is crucial for a number of reasons. First, it critically examines the western versus indigenous approaches and the dangers of the gulf between them as a matter of patients’ safety in treatment and rehabilitation. Second, it contributes to practice and policy by highlighting pragmatic responses to the problem which may require new practice and policy directions. Third, given the role of lack of access to and affordability of mental health care in the problem, it serves as a narrative on Nigeria’s level of commitment to sustainable development (1). Sustainable development goal 3 focuses on ensuring healthy lives and promoting well-being for all at all ages (20). In specific terms, it addresses mental health promotion (target 3.4) and treatment of mental disorders including substance use disorders (target 3.5). It also focuses on universal health coverage with an emphasis on financial risk protection as well as access to quality healthcare and affordability of medication. Notably, the indicators for universal health coverage would include essential health services coverage and proportion of household income expended on health. These two elements – care access and funding – are crucial considerations in mental health service planning and have a role to play in the utilization of indigenous mental health care in the country. Therefore, the current review examines the prospects and challenges in indigenous mental healthcare in Nigeria from a patient’s rights perspective while highlighting the role of culture and stigma in its utilization.

## 2. Framing the cultural syntonicity of indigenous mental health interventions

Culture refers to a complex aggregate of knowledge, belief, art, morals, law, custom and other social capabilities of a defined society. It is the shared knowledge required to function effectively in a given social system (21). Its significance in the clinical context is its relevance to the expression of distress and its explanatory function in illness causation, search for treatment and hope for recovery. Spirituality is a central feature of most non-western cultures (22) and Nigeria is not an exception. The dominance of western-style approaches to mental health may not be helpful in the appropriate conceptualization of indigenous methods of illness diagnosis and treatment in the long run in deeply cultural societies.

Cultural competence is central to navigating the critical junction between culture and mental illness as well as its treatment. A culturally competent approach to understanding the role of indigenous mental health care approaches within Nigeria will aim at situating them correctly in the realities of the patient. Cultural competence may be regarded as having the cultural knowledge and skills of a particular culture. Its aim is to ensure the delivery of effective interventions to members of that cultural group or identity (23). It basically comprises three essential skills: (i) scientific mindedness, (ii) dynamic sizing, and (iii) culture-specific expertise (23, 24).

Scientific mindedness describes the clinician’s capacity for developing hypotheses about the conditions of individuals from diverse cultural backgrounds. This prevents the clinician from drawing premature (and sometimes, wrong) conclusions about persons from different cultural backgrounds. With this perspective, scientific mindedness also helps the clinician to avoid the ‘myth of sameness’ (25). By testing hypotheses framed by existing cultural meanings attached to mental symptoms and available treatments, suitable inferences may be drawn by the “orthodox” practitioner about the patient’s view of their illness as well as their preferred indigenous approach to treatment in some instances. This systematic appreciation of the patient’s reality will help to form a partnership which will aid the patient to weigh the pros and cons of the preferred treatment narrative.

Dynamic sizing helps the clinician to know when generalization and inclusivity are relevant to members of a particular culture and when it is more appropriate to treat the patient as an individual who may be exclusive within his culture (23). Dynamic sizing seeks to prevent the emergence of stereotypes which directly or indirectly become the lenses through which the clinician views the individual and unconsciously ascribes to them the characteristics of their group of origin.

Culture-specific expertise essentially involves being aware of one’s worldview as well as having specific knowledge about the cultural groups to which the patients belong. It includes the ability of the clinician to adopt culturally-based or culturally appropriate approaches in treating patients.

Without paying due attention to cultural competence, our understanding of mental illnesses as well as their treatments may be unconsciously open to cross-cultural biases. A good example of such bias is seen in the African construct of mental disorder as being of a spiritual origin or supernatural intervention, which is in contrast to western biomedical conceptualizations of health (8, 10, 14, 26, 27). While the indigenous patient wishes to provide explanations for the disorder and its treatment using such spiritual perspectives, the clinician who is orthodox in their practice would conclude that such explanations are neither scientific nor helpful to the patient.

Within this context, there are two positions that practitioners can take in their appreciation of mental health interventions conceivable to the patient – a culture-blind approach or a culture-sensitive perspective. This culture-blind approach has been referred to as an ‘etic perspective’ (26) defined as the observing scientist’s conceptualization of a problem. The second position is attempting to examine the validity of the causation framework and treatment preference of the patient within a perspective of cultural relativity. This is the ‘emic perspective’ and it recognizes local cultural criteria for determining normative cultural expression.

The conceptualization of the cultural understanding of the personality of the African which is critically important in the cultural approach to mental illness and its treatment suggests that the African collective unconscious entails the self, spirit agency and social agency (28). The self includes the physical person, shadow, their clothing, as well as body fluids, amongst others. Spirit agency refers to multiple equal gods in the frame of polytheism in addition to ancestral and other spirits. The social agency comprises the extended family as well as the wider community.

Qualitative studies of mental health service users and non-orthodox treatment providers in African settings suggest that there is a prevailing perception of mental illness as being multifactorial with a dense interconnectivity between spiritual, biomedical and psychosocial etiologies (29, 30). Some of these causal ontologies may lead to stigmatizing attitudes (27). The existing perception leads to a form of help-seeking which combines these three approaches without mutual exclusivity since the different causal factors are deemed to require distinct but combinable treatments (30). A series of focus groups discussions among traditional healers in South Africa suggested that these indigenous healers demonstrate multiple explanatory models for mental disorders (31). While they view psychotic disorders as being prototypical of mental illness, they did not regard non-psychotic depression, panic disorder or somatization as mental disorders. There also appears to be a contemporary tendency among them of combining both traditional herbs and orthodox medicines in their indigenous interventions.

Against such a background, a preference for a spiritual formulation of mental disorders in an exclusive frame or within an eclectic combination (12) with biomedical causation would appear almost inevitable in deeply cultural settings. Due to such cultural predilections for an alternative formulation of mental illness apart from the biomedical explanation, patients and relatives may utilize indigenous services which may range from so-called ‘rehabilitation’ centers to spiritual homes and “trado-medical” healers. These centers are seen as treatment facilities for mental disorder or ‘correctional’/‘rehabilitation’ centers for drug addiction.

In these settings, the concept of mental disorder is not biomedical and is essentially related to moral and religious concepts of illness (27, 29). Under this rubric, practitioners of indigenous treatment methods usually institute ascetic approaches with spiritual undertones, e.g., fasting, beating with different ‘spiritually empowered’ objects, etc. in order to achieve physical restraint, symptom management or even cure.

## 3. The role of stigma

Stigmatization is a complex process which involves elements such as labeling, othering (“we vs. them”), discrimination and devaluation (32). It has also been constructed as a continuum from stereotypes through prejudice to discrimination (33). It may occur as public (33, 34), institutional (32, 35, 36) or internalized (15, 37) variants. Research in Nigeria has shown a considerable degree of both public and internalized stigmatization toward mental disorders (14, 15). Public stigma toward the mentally ill in the country has been found to be impelled by their perceived dangerousness, a spiritual view of causation of mental illness and social distance toward the mentally ill (14, 38).

Stigma may negatively affect treatment seeking, decisions regarding treatment as well as outcomes (39, 40). It may broadly affect access to and continuity of care thereby negatively impacting outcome. Catalano and colleagues have shown through structural equation modelling that patients’ awareness of negative stereotypes could influence them to agree with these stereotypes and self-apply them. This internalization may then lead to loss of self-esteem and poorer recovery attitudes. Fadipe et al. (40) and Adewuya et al. (41) have equally observed that self-stigmatization may be associated with poorer medication use.

The need to avoid stigmatization could lead to the utilization of indigenous or religious mental health services which are driven by a spiritual causation model – e.g. “spiritual attack” – which could make mental illness less stigmatizing (42). This is because the supposed attack is potentially curable by spiritual exorcism, propitiation or any other mystical means while the biomedical formulation may imply life-long manageable conditions which may not be amenable to cure making them more stigmatizing. Further, people may be reluctant to seek orthodox mental health care in order to avoid being labeled with a diagnosis of mental illness or due to a lack of trust in orthodox medicine (43).

Dealing with stigma will improve timely access to the right kind of treatment and result in better outcomes. A three-prong approach for dealing with stigma has been proposed by Corrigan & Watson (33, 37). These include education, contact and protest. Education is underpinned by public enlightenment to increase knowledge and ‘burst myths.’ Contact provides a link to the voices of sufferers with testimonies of their lived experience. This serves to disconfirm stereotypes, diminish anxiety, heighten empathy, create personal connections and improves our understanding of recovery. Protest is essentially advocacy which highlights and brings to the fore the challenges of those who live with mental illness and how society must show responsibility in looking after them while respecting their human rights.

## 4. Mental health treatment gap in Nigeria

The treatment gap for mental disorders in low and middle-income countries including Nigeria is up to 80% (18). Only 20% of those with severe mental illness in Nigeria have received any treatment in the preceding year and only 10% of those who received any treatment received minimally adequate treatment (4). Health expenditure toward mental health in Nigeria is about 3% as opposed to the recommended level of about 10% (44). Within the expenditure for mental health, a little over 90% is committed to eight specialist psychiatric hospitals which currently provide over 80% of the total number of psychiatric beds in the country which stands at 3.99 beds per 100,000. The remaining 9% of the provision for mental health is spent on other psychiatric services (university departments of psychiatry, other tertiary hospital settings, amongst others) (45). In terms of manpower, the World Health Organisation’s estimates of mental healthcare manpower for Nigeria reveals figures of 0.10/100,000 for psychiatrists, 0.70/100,000 for psychiatric nurses and 0.02/100,000 for psychologists, 0.04/100,000 for social workers and 0.01/100,000 for occupational therapists (46).

Within the context of the gaps in treatment occasioned by poor service planning, lack of manpower, and inadequate funding, traditional and faith healers are patronized for reasons of accessibility, affordability, and availability. Flexibility of the services, cultural acceptability as well as responsivity to cultural preference (42) may also play a role. Additionally, the absence of formal documentation may tap into the informality of confidentiality in African cultures. This helps to anonymize the patient to an extent and provide a sense of secrecy. The lack of such secrecy in orthodox settings where different aspects of the patient’s history must be documented by several members of the multidisciplinary team structure could result in institutional stigmatization within such settings (32, 36). Moreso, mental disorders unlike most physical health conditions do not have a clear-cut etiological agent or pathogenesis. This may feed the culturally acceptable stereotypes that mental illnesses are a product of divinity, witchcraft, immorality, and the influence of ancestral forces (47). It is therefore unsurprising that to tackle these spiritual forces, people may seek spiritual/indigenous care as part of their pathway to receiving treatment.

## 5. Three approaches of orthodox systems to indigenous mental health care

### 5.1. Orthodox dichotomization

Dichomotization is operationally described in this paper as the outright prohibition of non-orthodox practice or a dichotomization between the orthodox and the non-orthodox. This approach does not appear to be culturally sensitive in that it patently denies the emic perspective of the African patient in favor of an etic viewpoint that may not be sufficiently explanatory in dealing with psychological illness in the deeply cultural African patient (26). The idioms of distress are culturally framed (48–50) and provide a more accurate understanding of the patient’s psychological condition, especially as there are cultural beliefs around spiritual causes of mental health disorders (51).

Furthermore, while it may be argued that with increasing levels of literacy, the individual patient could have a personal preference for orthodox care, the communitarian ethos of Nigerian societies make it unlikely for the form of treatment for mental disorder to be decided by the individual alone but rather by the family within a communal consultative framework. Another key factor that influences the decision regarding the mode of treatment that patients receive is poor financial coverage for healthcare including mental healthcare in Nigeria with out-of-pocket being the commonest payment method (about 70.5% in 2019) (44, 52). Further, majority of patients are likely to depend on their relatives considering their relatively low socioeconomic status (53). Thus, they may have little or no say in deciding where and/or what type of care they access.

### 5.2. Interactive dimensionalization

A second response is that of interactive dimensionalization. This operationally refers to both forms of care occurring in a cultural continuum in which milder forms are treated in indigenous (unorthodox) settings while more severe cases are managed in psychiatric facilities. An inversion of the continuum seems to occur in rehabilitation. In that instance, those with acute “treatable” conditions are treated in orthodox settings while chronically ill patients are “rehabilitated” in these unorthodox settings. This raises the question as to whether this normative response is a psychosocial adjustment to shortages in healthcare planning and provision. This normative response is not unique to Nigeria, but also evident in the Western part of the world where indigenous treatment settings are not particularly prominent. Both clinicians and lay persons’ health and illness beliefs are influenced by knowledge, beliefs and attitudes (54). As such, causal ontologies of distress will be ingrained within individual and collective cultural values, which would have implications on decision making process and ethical principles with regards to patients’ autonomy, justice, beneficence, and non-maleficence (55–57). Therefore, interactive dimensionalization presents an opportunity for stakeholders, i.e., orthodox, and unorthodox practitioners, by moving mental health discourse away from dichotomization to exploring potential benefits of preventative and/or rehabilitative work, which would stem from paying closer attention to “existing assumptions about truth, validity and reality” [(58), p. 930]. Such exploration would for instance, accommodate patients’ preference for traditional healing (patient values and/or family members) or co-opt best empirical evidence available with clinical expertise of orthodox practitioners. Such operationalization of evidence-based practice could provide patients with much needed psychological safety at such a crucial time of their lives, which could be paramount for their personal recovery and ultimately, reduce stigma associated with mental health care pathways. It has been argued that patients in orthodox settings sometimes do not feel that their spiritual needs in treatment are recognized or met (18).

### 5.3. Collaborative shared care

A third dimension is collaboration which has now been termed “collaborative shared care” (4). This has been found to be associated with reduction in harmful practices in those non-orthodox healthcare systems while also resulting in improved clinical outcomes, reduction in disability as well as aiding reintegration (4). Further the evidence that traditional and faith healers may be willing to collaborate with orthodox practitioners is a strength of this dimension of care (4).

This potential collaboration between orthodox and unorthodox approaches in Nigeria demonstrates a preference for traditional and Christian religion-based healing (51). In this context, Wieringa et al. (58) argued that stakeholders would need to overcome the “philosophical problem of induction” by generally taking positive risks and making inferences that are context-driven even though, outcome might not always be positive. In the Nigerian context, this dimension allows stakeholders to showcase their knowledge and preferences, which will consolidate the idea that “bias is in a dual, complex, necessary, unproductive as well as a productive conjunction with truth” [(58), p. 936]. This philosophical stance therefore embraces a culture-conscious ideology, which requires a shift from solely acknowledging the dominant bio-medical model as the “truth,” to working in collaboration with interpersonal, socio-political, psychological, moral, and traditional frameworks.

Not acknowledging the inseverable link between truth and bias has contributed to dichotomization of orthodox and unorthodox practices. Therefore, this collaborative shared care dimension is of necessity for a country with over 300 ethnic communities as it would help recognize the value of indigenous approaches and interventions and by so doing, mitigate one form of exclusion from mental health care arising from the false distinction of what the “truth” is. For instance, it is often said that patients undergoing psychotic episodes “are not in touch with reality.” Taking this stance, such patients and/or family members’ needs, and preferences would likely be missed, resulting in iatrogenic harm. Treating patients who would likely pose serious risks to themselves, and others as *risky objects* would contribute to hermeneutical injustices, where subject (patients) would not be in the right frame of mind to adequately comprehend key components of what is being experienced, could be marginalized based on what is assumed by *others* not to be a reality and in turn, aggravating distress for subject (59). Mitigating hermeneutic and epistemic injustices on patients, a collaborative shared care dimension embedded within mental health care pathway has the propensity to mitigate defensive practices and promote defensible approaches, which would aid genuine “openness toward alternative horizons of possibility” [(59), p. 244]. Ultimately, this approach would help the ambition of World Health Organization (WHO) in delivering preventative work, restructuring and scaling up mental health care beyond inpatient clinical environments (60).

Nevertheless, it is crucial to examine the reciprocal perception between orthodox (biomedical) practitioners and indigenous treatment providers which may serve as barriers to collaboration. Recent qualitative research conducted in selected African countries indicates that there is mutual mistrust and undue competition between these two categories of carers (61, 62). While the traditional and religious healers have indicated a willingness to collaborate with orthodox practitioners in some cases, it would appear that the latter do not appreciate the skills/expertise of the former although this perceived superiority complex may be improved by dialogue and training (63). Where the orthodox practitioners have been willing to collaborate in a limited manner, they have often sought to impose supervision and control as well as training in biomedical paradigms on the indigenous treatment providers to their dissatisfaction (62).

In spite of the foregoing, it has been recognized that indigenous treatment practices tend to demonstrate inherent harmful effects such as shackling, beating, scarification (with the risk of serious infections), sexual abuse and adverse reactions from herbs which have hardly been pharmacologically tested for safety or adverse effect profiles (4). Any form of collaborative shared care involving indigenous practitioners must therefore clearly define how instances of harmful practices will be prevented, curtailed, or reported when they cross certain legal thresholds (e.g., sexual abuse) (4). Certainly, how this sort of safeguarding approach will affect such collaborative efforts remains to be seen.

## 6. Human rights in indigenous treatment settings

In non-orthodox treatment settings where the causation of mental disorder is regarded as being spiritual or moral, religious and/or moral as well as other cultural approaches are utilized for the purpose of treating the mentally ill. There may be a perceived need to introduce “disciplinary” methods or moral instruction coupled with punishment as part of the treatment. Ascetic methods including prolonged fasting, binding people in chains, sometimes in open spaces such as bushes. Such harsh treatments have been backed by relatives in some cases. It is within such environments that human rights abuses seem to occur.

Media reports from September to November 2019 in Nigeria revealed successive discoveries of such treatment centers across the country with troubling stories of torture and other forms of abuse [(17, 64); The Guardian Editorial (65)]. Those facilities were variously branded “torture houses” or “torture homes.” These reports indicated that some of these facilities were actually operating as religious centers which were then found to be housing several individuals with mental and substance use disorders admitted there for “treatment.” Over 1,200 persons were rescued from these facilities based on the intervention of law enforcement agencies. Another 200 individuals had been recorded as escapees from such treatment centers (see Table 1).

**Table 1:** Reported cases of individuals rescued from indigenous treatment settings in Nigeria.

Location	Number rescued	Year
Zaria, Kaduna	11	2019
Rigasa, Kaduna	Approx. 300	2019
Daura, Katsina	360 + 200 escapees	2019
Rigasa, Kaduna	147	2019
Gaa-Odota, Kwara	108	2019
Yola, Adamawa	15	2019
Ibadan	259	2019

Authors’ compilation from media reports in Nigeria from September 30, 2019 to November 3, 2019.

Graphic images released by the media at the point of rescuing the victims indicated unsanitary conditions, physical health debilitation, and evidence of mechanical restraints used in crude and harmful ways (17). The Human Rights Watch had equally reported that patients within such settings have been prone to being shackled or chained [Human Rights (16)].

### 6.1. Indigenous mental health treatment and torture: A medico-legal viewpoint

#### 6.1.1. Legal perspective

Torture has been globally defined as an act by which severe physical or mental pain or suffering is intentionally and unlawfully inflicted on a person by or at the instigation of public officials or others acting in any official capacity for such purposes as obtaining a confession, intimidation, punishment, undue coercion and other reasons based on discrimination of any kind (66). The Nigerian Medical community has equally described torture as a systematic infliction of physical and/or mental injury which is harmful on a person by others for any reason which undermines personal dignity (67). Section 2 of the Nigerian legislation against torture also defines it in similar terms [Anti-torture (68)].

Physical torture involves beating, punching or slapping, suspension of body frames in unusual positions, sexual torture, rape, forceful insertion of objects into body orifices, tearing, torching or burning or exercises other than usual training procedures, and climate stress such as application of extremes of heat or cold, amongst others. Biological and chemical torture may be pharmacological, which is misuse of indicated and unapproved drugs; forced urine and excrement usage, and application, sleep deprivation, starvation, insect or animal aggression, in addition to other forms. Psychological torture could involve threats to self and loved ones, sexual violations, deprivation of healthcare comfort to either the victim or his family, and forced witnessing of the torture of others. According to media reports of victims’ accounts, forms of torture in these indigenous centers included starvation, beating, the use of chains or shackles, hanging, sexual abuse including rape and sodomy as well as other forms of abuse, exploitation or degrading treatment.

A number of important legal instruments are relevant to torture in any context. Article 3 of the Universal Declaration of Human Rights (UNDHR) (69) and article 6 of the African Charter on Human and Peoples’ Rights (ACHPR) (70) clearly state that everyone has the right to life, liberty and security of person while Article 5 prohibits a person being subjected to torture or to cruel, inhuman or degrading treatment or punishment. Similarly, article 12 of the ACHPR (1986) and article 13 of the UNDHR (1948) guarantee freedom of movement which is consistent with section 35 of the Nigerian constitution.

In the same vein, criminal law sanctions exist against torture within the shores of Nigeria. Section 8 of Anti-torture Act (68) makes participation in torture a crime and it would appear that this may make the relations of torture victims liable as conspirators or accessories after the fact when they become aware that their wards are being tortured and they fail to report it. Section 9(1) of the Anti-torture Act (68) imposes a jail term not exceeding 25 years on offender convicted for torture of any form/kind while section 9(2) of the same act prefers a charge of murder against an individual who tortures a person to death. Instructively, section 9(3) of the Anti-torture Act (68) provides for civil suit (human rights) against the perpetrator regardless of criminal proceedings. This will be helpful for recovering necessary damages in cases of torture thereby further assuaging the victim’s sense of loss.

#### 6.1.2. Ethical perspective

From an ethics point of view, it is important to stress the divergent ethical positioning of orthodox mental health practitioners from those of indigenous treatment providers. While the physician has the professional ethical as well as moral duty to be beneficent, non-maleficent, just and respectful of individual autonomy (67, 72), these loosely regulated alternative treatment centers have no such professional ethics. They espouse the ethos of power over the vulnerable for his/her own benefit which is essentially a patent mix of paternalistic and humanitarian tendencies. Within the African collective unconscious, they frequently recognize the role of the spiritual in disease causation. They thus see the need to wield spiritual power over etiological demons/evil spirits and such powers can be exercised in whatever way is randomly possible without recourse to the patient’s human rights or personal dignity. It is noteworthy that some of these unethical practices have surprisingly been reported in orthodox treatment settings (12). This may not be unrelated to the absence, for many years, of enabling laws that protect persons with mental-ill health thus necessitating a need for prompt review of the obsolete mental health laws that currently exist in Nigeria in order to stem these unwholesome practices. A new legislation has now been passed and it substantially promotes the rights of persons with mental disorders (77).

Furthermore, the doctor must not be party to torture [(67); rule 66.0]. While this should guide the physician against engaging torture techniques in treatment, it also raises concerns when one considers that in a collaborative shared mode of care, there will be a need to form a partnership of a sort with indigenous treatment providers. This remains an ethical concern which must be addressed transparently in any shared care arrangements with non-orthodox practitioners (4). Overall, a decent society has a moral duty to prevent torture and to protect its citizens from harm in a broad sense since, in the Nigerian context, ‘welfare and security’ is regarded as the primary purpose of government [(71); s. 14(2) (b)].

#### 6.1.3. Addressing the biopsychosocial impact of torture in indigenous mental healthcare

The effects of torture and social isolation in unregulated indigenous treatment centers are multi-faceted. The physical effects include malnutrition, infections (TB, HIV/AIDS/STIs, Hepatitis B, etc.), as well as untreated medical conditions such as hypertension, diabetes mellitus, asthma, dermatological conditions, etc. and in extreme instances, death may occur. The psychological sequelae of torture could potentially include mood disorders, psychosis, PTSD, generalized anxiety disorder, phobias, enduring personality change, sleep disorders, organic psychotic disorders from head injuries, persisting substance use disorders given the lack of effective treatment for years, later substance use disorders in order to cope with the effects of the trauma, adjustment disorders, shame, low self-esteem, and suicide, amongst many others. On the social level, the adverse effects of social isolation include loss of social and occupational skills, loss or lack of accommodation, loss of employment, marital difficulties, disruption of family ties, etc.

In rehabilitating torture victims or persons rescued from poorly managed indigenous treatment facilities, there should be a clear focus on physical health and psychosocial well-being. Victim assistance will include screening for physical health problems, e.g., HIV, Hepatitis, etc., post-trauma psychological intervention, e.g., counseling and perhaps relevant treatment for mental health problems/drug addiction which brought the individuals into such facilities in the first place. These rehabilitation objectives will require adequate reintegration of these persons into society including a focus on occupational rehabilitation. This will involve adequate inter-sectoral collaboration comprising the ministries of health, women and social welfare and justice. The involvement of their family members will always be critical. In order to ensure the success of such rehabilitation and reintegration strategies, a multi-disciplinary approach should be adopted. Responsible media coverage/reportage is equally important in ensuring the informational/spatial integrity of victims/patients in order not to further stigmatize them.

## 7. Managing the treatment gap as a means of dealing with harmful forms of indigenous care

Addressing the treatment gap in Nigeria will ensure a reduction of patients’ reliance on some indigenous forms of care which are harmful as well as lead to improvement in resources to collaborate more effectively with complementary and traditional care approaches that are beneficial. To achieve this treatment gap intervention, a number of initiatives must be taken. First, there must be better funding for healthcare with a focus on allocative efficiency. Second, community mental healthcare must be strengthened as a way of improving access, affordability, availability and responsivity to the preferences of patients. Within the community model of care, the integration of mental health into primary health care presents one of the most cost-effective approaches to care in low and middle-income countries. Early experiences in Nigeria show that its uptake is quite significant and its benefits are demonstrable (73). It reduces the social distance toward the mentally ill and mental health generally. It is to be noted that primary mental health care is likely to work best when broad structural barriers to health such as low levels of education, unemployment, poverty and key infrastructural deficits, e.g., transportation, are addressed (74).

Additionally, attempts must be made, through public enlightenment and other stigma-reduction strategies, to present a more positive view of already “stigmatized” psychiatric hospitals which continue to provide over 80% of the mental health beds in the country. There may come a time when most services will be decentralized to smaller, more community-based centers which could be more cost-effective (75) but at the moment, the specialist psychiatric services still offer the much needed care which the populace requires. Beyond the de-stigmatization of the stand-alone psychiatric hospitals, there is also a need for public enlightenment regarding the cause, symptoms and course of mental disorders which will serve to combat self- and public-stigma as well as social distance toward persons with mental illness (38). The role of advocacy in this regard by both governmental and non-governmental actors cannot be over-emphasized (33). The current mental health policy in the country is geared, among other things, toward the elimination of stigma related to mental illness by improving community awareness of mental health issues through evidence-based messages in the media and leveraging community support systems (76). The latest mental health legislation also addresses stigma and discrimination toward persons with mental disorders through a robust institutional framework [National Mental Health Act 2021 (77)].

Political will on the part of government is central to achieving these initiatives. Government must have the vision of increasing funding for mental health care from the paltry average of about 1% seen in LMICs (about 3.3% in Nigeria) to levels closer to the recommended level of about 10% of government health expenditure (44, 78) Currently, less than 5% of mental health research funding is made available to Low Middle Income Counties (LMICs) (60). Going forward, there needs to be an increase in clinical and applied research outputs from LMICs which would inform conceptual or theoretical models of care and underpinned by the understanding of interdependence self-construals.

Furthermore, there is a need to ensure the investigation and prosecution of offending indigenous treatment ‘facilities’ who abuse patients’ rights and jeopardize their safety (4). In this connection, the effective implementation of the recently enacted of the National Mental Health Act (77) which has provisions for the registration, licensing and accreditation of mental healthcare facilities is critical to exercising appropriate oversight in mental health care in the country. The force of statute will further strengthen law enforcement agents to apprehend indigenous mental health care providers going beyond the remit of providing culturally appropriate and acceptable care to operating as unlicensed psychiatric centers where the rights of patients are frequently abused.

In a more nuanced view of the role of spirituality and culture in mental health treatment, reliable research evidence reviewed in this paper suggests that there is demonstrable benefit in collaborating with traditional/faith healers/alternative mental health service providers within an appropriate framework while denouncing/addressing some of their harmful practices. This approach would aid a paradigm shift, with a focus on knowledge creation with psychotic patients for instance, being active agents, who are making sense of their reality (79) as supposed to discounting meanings associated to their experiences, which often result into restrictive and unethical practices. Future research needs to explore the application of phenomenological psychopathology within collectivist societies like Nigeria, where the construct of selfhood lays emphasis on relationships with others and are concerned with what their social groups think of them (80, 81). This has implications on day-to-day practices and would present itself when unwell. In the context of schizophrenia and related psychoses for instance, it is important to understanding stressors and coping strategies adopted by patients and their family members with the view of denouncing the convenience of dichomotization, which tends to oversimplify non-orthodox but evidence-based health practices. Additionally, knowledge sharing and on-going dialogue between orthodox and indigenous mental healthcare providers will go a long way to ease the tension between them and create greater opportunities for collaboration (62, 63).

Despite the important issues raised in this review, it is necessary to highlight a few notable limitations to our narrative approach. Since this was not a systematic review of the literature, it is likely that some important studies on the critical intersection between indigenous mental healthcare, cultural factors and stigmatization as a psychosocial response to mental illness in Nigeria could have been missed. Additionally, the media reports on inhumane treatment of the residents of some of the traditional or religious treatment facilities were selectively based on facilities that had attracted the attention of law enforcement agents. Generalization of the nature and severity of such human rights abuse to all traditional or religious mental health treatment settings must be done with caution. Evidently, the international conventions, criminal law legislations and medical ethics code referenced in this narrative review were those deemed by the authors to be most relevant to our discourse and cannot be said to be exhaustive. Future reviews could address these limitations by adopting a systematic review methodology using more streamlined research questions derivable from our narrative review. Finally, the authors being of an orthodox persuasion, may not be entirely value-free or neutral in the evaluation of the literature or the arguments proposed.

## 8. Conclusion

Indigenous mental healthcare is endemic in Nigeria with its complex underpinnings of stigma and cultural syntonicity as well as its inherent proclivity for human rights deprivations. Be that as it may, orthodox dichotomization as a dominant systemic response to it is unlikely to produce a meaningful care response. While interactive dimensionalization provides orthodox practitioners and policy makers with a realistic psychosocial explanation for the utilization of this variant of mental healthcare, collaborative shared care involving measured collaboration between orthodox mental health practitioners and indigenous mental health systems offers an effective as well as cost-effective intervention strategy. It reduces harmful effects of indigenous mental healthcare including human rights abuses and offers patients a culturally appropriate response to their problems (12). Overall, practical considerations suggest that indigenous mental health treatment providers have larger capacity for residential care (18), are perceived to be effective (82), and not likely to ‘go away’ (83). Although these complementary/alternative mental health service providers fall short of certain ethical and/or moral standards and could be more prone to the patients’ human rights abuses, they cannot be ignored and ought to be harnessed responsibly within this collaborative shared care framework. This does not only provide a pragmatic response to resource constraints within the mental healthcare environment in Nigeria but offers a cost-effective approach to safely meeting the health needs of the populace in meeting sustainable development targets in the frame of universal health coverage.

## Author contributions

AO conceptualized the review and wrote the first draft of the manuscript. BF contributed critical content on the aspects of stigma, indigenous mental healthcare utilization, and collaborative shared care. OB contributed critical content in elucidating the philosophical/ethical basis of interactive dimensionalization and collaborative shared care. All authors contributed to the article and approved the submitted version.

## Funding

The authors thank Liverpool John Moores University, UK for funding the processing fees of this article.

## Conflict of interest

The authors declare that the research was conducted in the absence of any commercial or financial relationships that could be construed as a potential conflict of interest.

## Publisher’s note

All claims expressed in this article are solely those of the authors and do not necessarily represent those of their affiliated organizations, or those of the publisher, the editors and the reviewers. Any product that may be evaluated in this article, or claim that may be made by its manufacturer, is not guaranteed or endorsed by the publisher.
